# Cold Atmospheric Plasma: Possible Cure of Autoimmune Disorders and Cancer via Attenuating Inflammation

**DOI:** 10.7150/ijbs.102445

**Published:** 2024-10-07

**Authors:** Xiaofeng Dai, Shuo Feng, Yan Zheng

**Affiliations:** 1 National Local Joint Engineering Research Center for Precision Surgery & Regenerative Medicine, Shaanxi Provincial Center for Regenerative Medicine and Surgical Engineering, the First Affiliated Hospital of Xi'an Jiaotong University, Xi'an 710061, P.R. China; 2 Department of Dermatology, the First Affiliated Hospital of Xi'an Jiaotong University, Xi'an 710061, P.R. China.

**Keywords:** Autoimmune disorder, Cancer, Inflammation, Cold atmospheric plasma, Macrophage polarization, Mitochondria

## Abstract

Autoimmune diseases and cancers, two seemingly unrelated diseases, have been threatening human health, and many of them have no cure. By identifying pathological inflammation as the driving cause of uncontrolled cell proliferation in both classes of diseases, and differentiating autoimmune disorders and cancers by whether the cell death programs are under control, we propose the attenuation of prolonged inflammation via maintaining mitochondrial reduction-oxidation (redox) homeostasis being a possible cure of both diseases. Importantly, we propose the feasibility of applying cold atmospheric plasma (CAP) in treating autoimmune disorders and cancers given its redox-modulatory nature, which not only extends the medical utilities of CAP to autoimmune diseases and all other inflammation-driven disorders, but also positions the efficacy of CAP against cancer cells to its suppressive role on prolonged inflammation. Our insights may open an innovative avenue towards a unified view on the molecular mechanism driving the diversified types of medical miracles of CAP and what CAP can do in the field of plasma medicine.

## Introduction

Immune system disorders are caused by abnormally low or high activity of the immune system. While immune deficiency renders the body vulnerable to infections, immune over-activity is typically associated with autoimmune diseases as antibodies produced by the immune system attack and damage its own tissues instead of fighting against infections, the process of which develops inflammation. Typical therapeutic strategies for autoimmune disorder control are based on systemic immunosuppression that, although ameliorate symptoms, are typically associated with long-term adverse effects and require continuous oral or injectable medications that imposes economic burden to the patients. Thus, despite current medication advances, cure of autoimmune diseases remains elusive, and patients still suffer from progressive disability with shortened life span and comorbidity due to, possibly, weakened immunity.

Cancers can be considered as chronic metabolic and immune disorders featured with 10 hallmarks that can be summarized to 'uncontrolled proliferation', 'evading programmed cell death', 'tumour angiogenesis and metastasis', 'reprogrammed metabolism', 'genome instability', 'avoiding immune destruction', and 'tumour-promoting inflammation' 1. Among these features, 'tumour-promoting inflammation' together with 'genome instability' are considered enabling characteristics. While many targeted therapeutics aiming to suppress oncogenes and restore the activity of tumour suppressors towards enhanced genome stability have been launched on the market, they suffer from, e.g., unavoidable adverse effect and limited therapeutic spectrum, that render the call for novel onco-therapeutic design pertinent and urgent.

**Figure 1:**
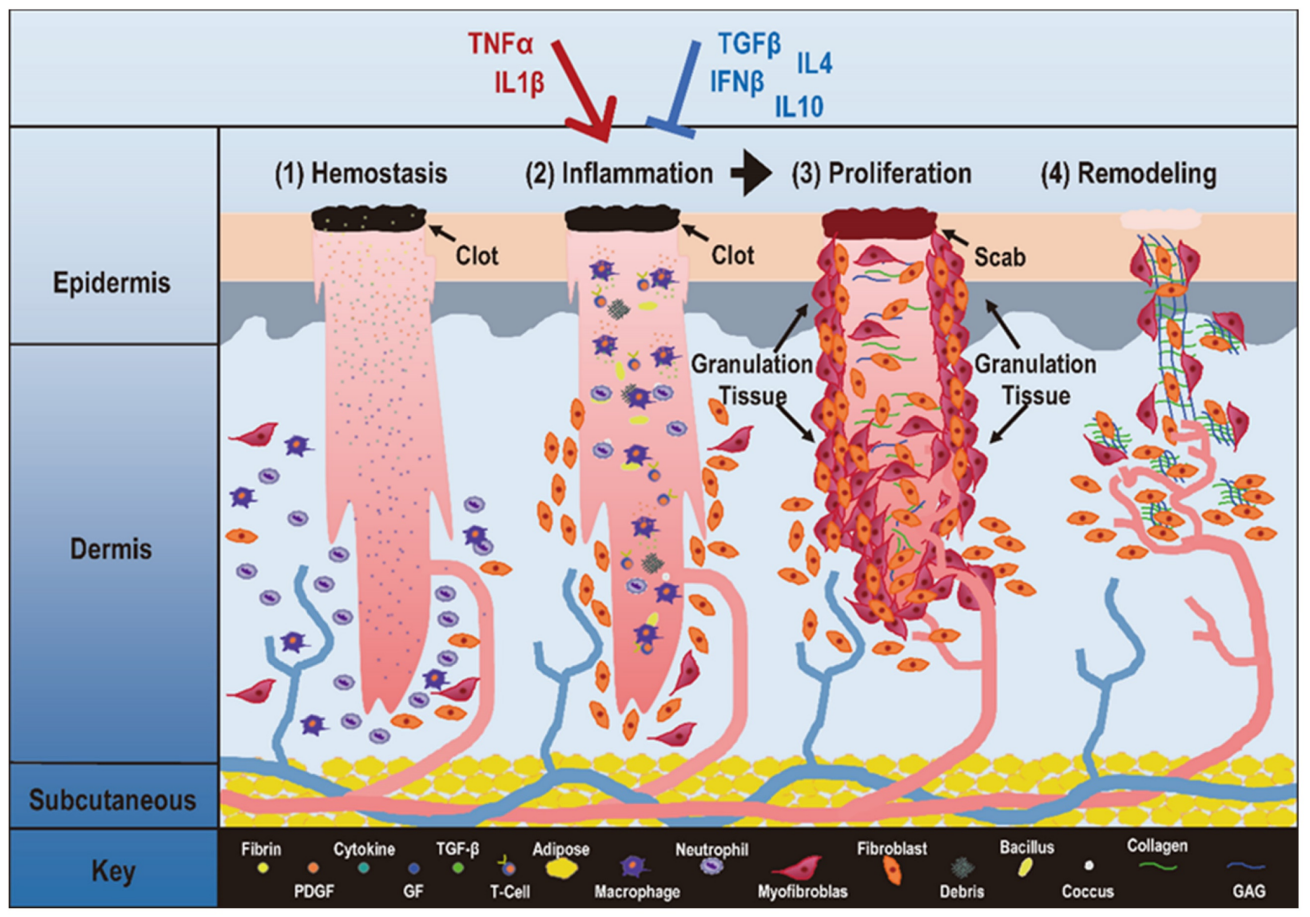
** Critical stages during physiological inflammation and its associations with autoimmune disorders and cancers.** Take 'wound healing' as the example, 'hemostasis', 'inflammation', 'proliferation', 'remodeling' are four stages that occur in the proper sequence and time frame. The first stage in wound healing is 'hemostasis' that can last for two days. During this stage, blood vessels constrict to reduce the blood flow (known as vasoconstriction), and clotting factors are released at the wound site to coagulate with fibrin and form a blood clot that can act as a seal between the broken blood vessels to prevent blood loss. The second phase of wound healing is 'Inflammation' that can last for 7 days or longer, and involves phagocytic cells that release ROS. During this phase, white blood cells and some enzymes enter the wound area to remove infection. The third phase is 'proliferation' that can last for 4 days to up to 3 weeks or more. In this stage, inflammatory cells undergo apoptosis, granulation tissue forms, angiogenesis starts, wound contracts, and epithelialization occurs. The fourth phase is 'remodeling' that may occur over months or years. During this phase, the new tissue gradually becomes stronger and more flexible, and collagen production continues to build the tensile strength and elasticity of the skin. An improper transition from inflammation to proliferation may lead to pathological consequences. Specifically, when pro-inflammatory cytokines such as IL1β and TNFα take a dominant role over anti-inflammatory cytokines such as TGFβ, IL10, IL4 and IFNβ, inflammation is prolonged that can lead to uncontrolled proliferation, a typical manifestation of many diseases including autoimmune diseases and cancers. This figure is redrawn and adapted under the terms and conditions of Creative Commons Attribution (CC-BY) license from 106.

Autoimmune disorders and cancers are two faces of aberrant immune responses. That is, the immune system mistakes one's own tissues as foreign invaders in autoimmune diseases, but misses the actual attack on malignant cells in cancers. Interestingly, disorders associated with false positive (autoimmune diseases) and false negative (cancers) immune responses are both featured with abnormal inflammation, rendering attenuation of pathological or prolonged inflammation a promising remedy for maintaining immune-homeostasis and a possible solution for resolving both types of diseases (**Figure 1**).

By interrogating the molecular cause and cellular consequence of inflammation, we identify mitochondrial net reactive oxygen species (ROS) as the recruiter of cytokines to the inflammatory loci and abnormal M1 macrophage polarization as the driving force of pathological inflammation that leads to uncontrolled cell proliferation, and differentiate autoimmune diseases and cancers by cells' ability in breaking the control on programmed cell death. Importantly, we emphasize the importance of maintaining reduction-oxidation (redox) homeostasis in preventing disorders including autoimmune diseases and cancers by attenuating inflammation, and forecast the possible use of cold atmospheric plasma (CAP) in treating such diseases using psoriasis and melanoma as the examples.

## Redox homeostasis is vital in maintaining normal cellular functionalities

The occurrence of many physiological events such as cell signaling and protein folding depends on redox homeostasis, and many pathological conditions have been associated with redox imbalance 2, 3.

Redox balance is critical for proteins to pertain normal functionalities, as increased levels of oxidants for a certain duration may oxidize spatially adjacent -SH groups to disulfides that possibly affect protein activities. For instance, the antioxidant protein thioredoxin 1 (TRX1) is enzymatically active if harboring two -SH groups at cysteine (Cys)32 and Cys35, but becomes inactivated if -SH groups at these two positions were oxidized to disulfides 4, and increased TRX1 protein oxidation was observed in more invasive human prostate cancer cells 5. However, while the development of human prostate cancer cells favors an oxidizing environment, a reducing condition was found in human breast cancer tissues 6, suggesting the importance of redox equilibrium in maintaining the healthy state of cells. Redox fluctuation has also been reported to affect protein functionality through affecting protein post-translational modifications (PTMs) such as phosphorylation, methylation, acetylation, sumoylation, nitrosylation, nitration and glutathionylation. For instance, enhanced ROS has been shown to perturb the gene expression profile of nuclear factor erythroid 2-related factor 2 (NRF2), a transcription factor (TF) of glutathione peroxidase 4 (GPX4) and the primary master of cellular redox homeostasis 7, 8.

Redox equilibrium may affect the stability of proteins, as an oxidizing condition favors disulfide bond formation towards enhanced protein stability and a reducing environment favors -SH groups 9. For instance, NRF2 is ubiquitinated under normoxic conditions, but stabilized in response to oxidative stress towards accelerated cancer progression 10.

Redox homeostasis is vital in regulating the transcription of genes responsible for redox stress adaption, as redox-sensing Cys residues are often located within the region required for DNA binding of redox-regulated TFs. For instance, the promoter regions of redox-regulated genes typically contain an anti-oxidant responsive element (ARE), a cyclic AMP responsive element (CRE), and specificity protein-1 (SP-1) sites required for activating TFs such as AP-1, nuclear factor kappa B (NFκB), NRF2, TP53 11, 12.

## Mitochondria net ROS plays pivotal roles in regulating redox homeostasis

Redox homeostasis is dynamically regulated through the glutathione and thioredoxin systems that serve as two parallel yet non-redundant axes to allow sufficient vulnerability of cells to respond to cellular stress. While the glutathione system is defined as the relative concentrations of glutathione (GSH) and its oxidized, disulfide-bonded dimer glutathione disulfide (GSSG) 13, 14, the thioredoxin system refers to the cysteine/cystine (Cys/Cyst) couple.

**Figure 2:**
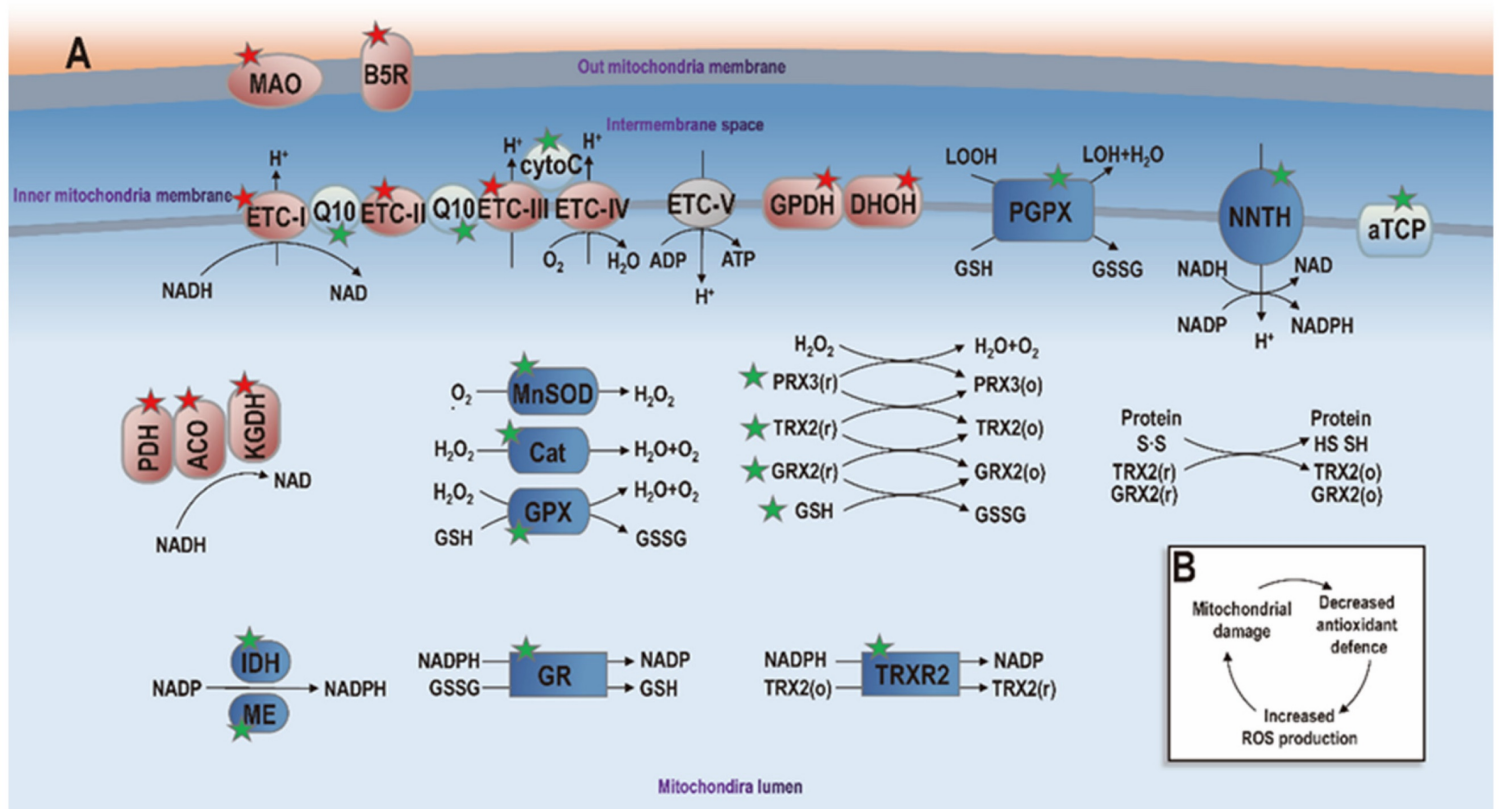
** Diagram illustrating mitochondria redox homeostasis.** Events involved in ROS generation and anti-oxidant defense are marked with red and green stars, respectively. Proteins taking part in pro- and anti-oxidant events are symbolized in 'red' and 'blue', respectively. Proteins playing non-oxidative roles are marked with 'grey'. Mitochondrial enzymes known to generate ROS include pyruvate dehydrogenase (PDH), aconitase (ACO), α-ketoglutarate dehydrogenase (KGDH), electron transport chain complex I (ETC-I) to III (ETC-III), glycerol-3-phosphate dehydrogenase (GPDH), dihydroorotate dehydrogenase (DHOH), monoamine oxidase (MAO), cytochrome b5 reductase (B5R). When these enzymes are abundantly charged and the mitochondrial membrane potential is high, electrons are transferred to oxygen towards ROS generation. Mitochondria also contain a large anti-oxidant defense system to maintain the ROS balance in mitochondria. Non-enzymatic components of this anti-oxidant system include α-tocopherol (αTCP), coenzyme Q10 (Q10), cytochrome C (cytoC) and glutathione (GSH), which are symbolized using 'light blue'. Enzymatic components include manganese superoxide dismutase (MnSOD), catalase (Cat), glutathione peroxidase (GPX), phospholipid hydroperoxide glutathione peroxidase (PGPX), glutathione reductase (GR), peroxiredoxins (PRX3/5), glutaredoxin (GRX2), thioredoxin (TRX2), and thioredoxin reductase (TRXR2), isocitrate dehydrogenase (IDH), malic enzyme (ME) and nicotinamide nucleotide transhydrogenase (NNTH), which are represented in 'dark blue'. When mitochondria redox homeostasis is perturbed, net ROS is produced that can damage mitochondria, leading to decreased anti-oxidant ability and increased ROS generation that can further damage mitochondria (panel B). Additional annotations: GSSG, glutathione disulfide; LOH, lipid hydroxide; LOOH, lipid hydroperoxide; o, oxidized state; r, reduced state. This figure is redrawn and adapted with permission from 13.

Redox homeostasis is regulated at the level of subcellular compartments, among which mitochondria is the most reducing and thus most susceptible to oxidation in response to cellular stress 15. Thus, mitochondria perform multiple essential tasks that depend on redox modulation such as energy production for cell proliferation and death signal sensing for cell fate determination. As the primary cellular oxygen consumers, mitochondria contain numerous enzymes capable of transferring single electrons to oxygen towards the production of superoxide anion (O_2_^-^), one key type of reactive oxygen and nitrogen species (RONS). These redox enzymes include, e.g., aconitase (ACO) and α-ketoglutarate dehydrogenase (KGDH) from the tricarboxylic acid (TCA) cycle, the electron-transport chain (ETC) complexes I to III, pyruvate dehydrogenase (PDH) and glycerol-3-phosphate dehydrogenase (GPDH), dihydroorotate dehydrogenase (DHOH), monoamine oxidases A and B (MAOA/B), and cytochrome *b*_5_ reductase (B5R) 13 (**Figure 2**). The transfer of electrons to oxygen is more likely to occur when these redox enzymes are charged with sufficient electrons and have high energy transfer potential or, in other words, high mitochondrial membrane potential.

Mitochondria also contain an extensive antioxidant defense system to detoxify generated ROS that are tied to the redox state of mitochondria. The antioxidant defense system is comprised of non-enzymatic and enzymatic components. While non-enzymatic components include α-tocopherol (aTCP), coenzyme Q10, cytochrome C, and GSH, recognized antioxidant enzymes include manganese superoxide dismutase (MnSOD), catalase (Cat), GPX4, phospholipid hydroperoxide glutathione peroxidase (PGPX), glutathione reductase (GR), peroxiredoxins 3/5 (PRX3/5), glutaredoxin 2 (GRX2), TRX1 and thioredoxin reductase 2 (TRXR2) 13 (**Figure 2**).

When the mitochondria are structurally and functionally intact, little net ROS is produced given its effective antioxidant defense system that balances out generated ROS. When the antioxidant defense system of the mitochondria is impaired, net free radicals are generated that can further damage mitochondria, leading to a vicious cycle and, consequently, an increasing amount of transferable electrons and various forms of ROS. For example, the Fe-S cluster in ACO is easily inactivated by O_2_^-^, and the iron is released once this occurs, leading to the production of hydroxyl radical (‧OH), O_2_^-^, singlet oxygen (^¹^O_2_), and peroxynitrite (ONOO^-^), which further amplify oxidative damage, all of which are important ROS.

### Redox disequilibrium triggers pathological inflammation

### Cytokines are primary mediators of inflammation

Inflammation refers to a response to external or internal stimuli that results in tissue repair under the physiological condition or undesirable immune responses under the pathological condition 16. Cytokines and chemokines, among other factors, are known to mediate inflammation. While chemokines such as chemokine (C-X-C motif) ligand 8 (CXCL8) are small cytokine-like proteins activating immune cells during inflammation and attracting them to the sites of inflammation, cytokines play the primary roles 17.

Cytokines can be pro-inflammatory or anti-inflammatory. Typical pro-inflammatory cytokines include, e.g., interleukin 1β (IL1β) and tumor necrosis factor α (TNFα), and function by inducing adhesion molecules and chemokine expression that recruit leukocytes 18. Specifically, IL1β is a potent activator of leukocytes capable of enhancing the killing efficacy of NK cells and macrophages, inducing T cell proliferation and B cell antibody production, and triggering the expression of additional cytokines such as TNFα, IL1β, IL2, IL3, IL6, and interferon γ (IFNγ). TNFα is an inducer of inflammatory mediators such as IL1β, IL6, granulocyte-macrophage colony-stimulating factor (GM-CSF) 19, 20, chemokines, and adhesion molecules, and is a recruiter as well as a stimulator of leukocytes to the site of inflammation for cytokine production 21, 22 and ROS generation. Anti-inflammatory cytokines include TGFβ, IL10, IL4, and members of type I IFNs especially IFNβ. One typical mechanism causing the anti-inflammatory properties of these cytokines is their down-regulatory effects on the activating endothelial cells and M1 macrophages. For instance, IFNβ antagonizes the effects of IL1β in several systems including the activation of M1 macrophages and IL6 induction 23. IL4 was found to reduce the release of IL12, a cytokine critical for inducing T helper 1 (Th1) response. IL10 is an inhibitor of activated M1 macrophages that counteract the effects of IFNγ. IFNβ blocks endothelial cell activation mediated by pro-inflammatory cytokines 24.

### Redox disequilibrium triggers pathological inflammation via macrophage M1 polarization

Physiological inflammation is self-limiting, whereas pathological inflammation runs out of control on NFκB signaling and Nod-like receptor family pyrin domain containing 3 (NLRP3) inflammasome, two archetypal drivers of the inflammatory response 16.

**Figure 3:**
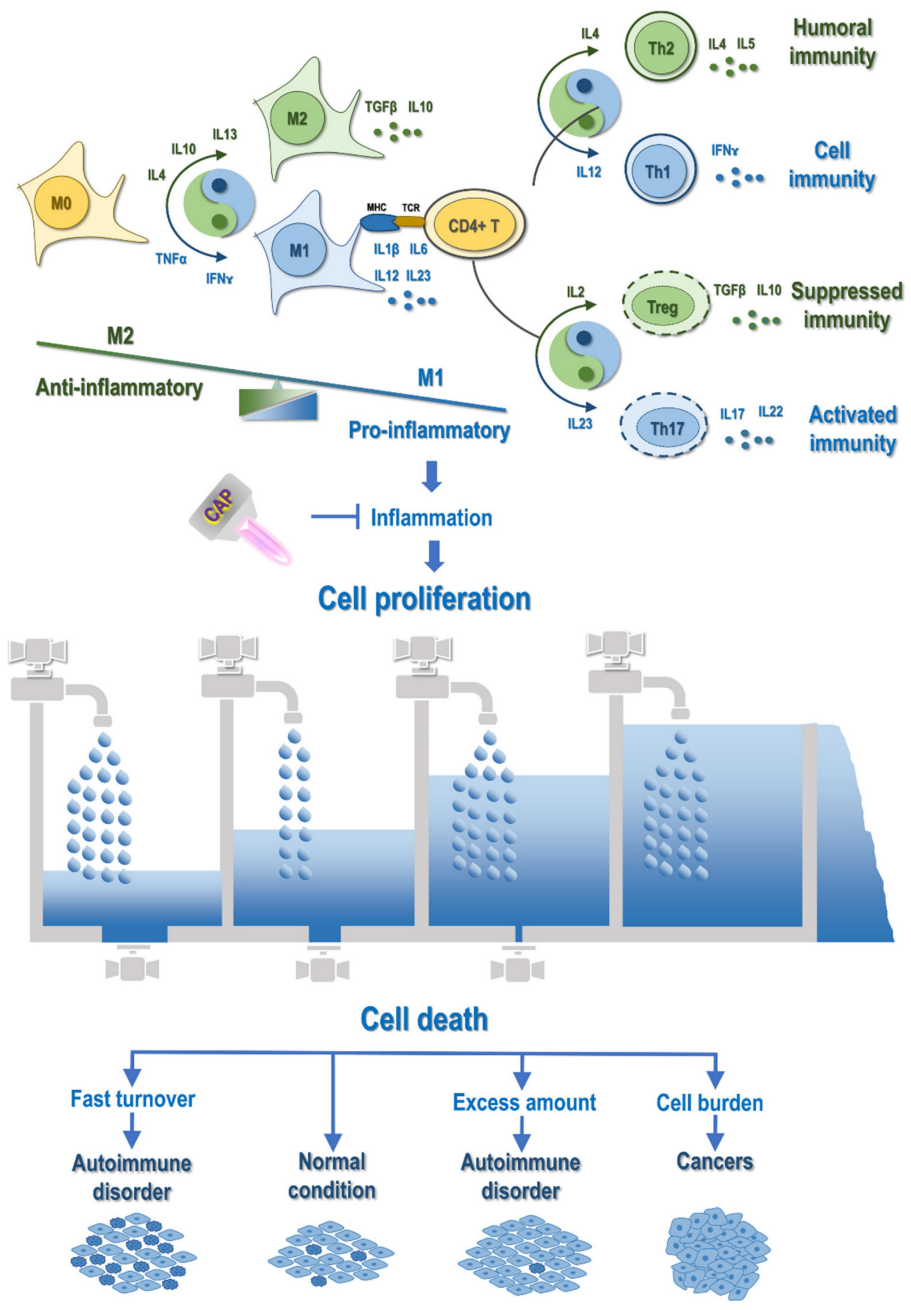
** Illustration on the mechanistic cause of autoimmune disorders and cancers and their differentiation regarding the life/death control.** Abnormal M1 macrophage polarization, among other possible mechanisms, promotes cell inflammation, with cytokines being the primary mediators. Primary cytokines capable of priming M1 polarization are TNFα and IFNɤ, and major cytokines triggering M2 differentiation are IL4, IL10 and IL13. M1 macrophages secrete cytokines such as IL1β, IL6, IL12, IL23, and M2 macrophages produce cytokines such as TGFβ and IL10. Activated macrophages present antigens to CD4+ T cells to activate the adaptive immune system, where cytokines play vital roles in modulating the distribution of T cell sub-cohorts. Primarily, IL12, IL4, IL23, IL2 trigger T helper 1 (Th1), Th2, Th17 and T regulatory (Treg) cell polarization, respectively, which produce IFNɤ, IL4/5, IL17/22, as well as TGFβ and IL10. Cytokines are the primary mediators of the interplay between the innate and adaptive immune responses. For example, both Treg and M2 produce TGFβ and IL10 that are associated suppressed immunity; Th17 is associated with activated immunity that can be primed by IL23, a cytokine secreted by M1; Th1 secretes IFNɤ that primes macrophages toward the M1 state; and Th2 produces IL4 that potentiates macrophages toward the M2 state. Prolonged inflammation causes uncontrolled cell proliferation, which leads to autoimmune disorders or cancers depending on, e.g., whether programmed cell death is under control. In this diagram, the inflow represents 'cell proliferation' and the outflow symbolizes 'cell death'. When both cell growth and cell death are accelerated as compared with that under the normal condition, cells are under fast turnover, leading to autoimmune disorders such as type I diabetes and multiple sclerosis. When cell growth is increased but cell death is decreased, excess cells are accumulated, leading to autoimmune diseases such as psoriasis and autoimmune lymphoproliferative syndrome. When cell growth is out of control and cell death is evaded, cell burden forms that can even migrate to other places, forming cancers. CAP can be a possible cure of autoimmune disorders and cancers by attenuating inflammation.

Endogenous ROS have been proposed capable of regulating immune cell functionality, proliferation and differentiation, including macrophage polarization. Macrophages can be pro- or anti-inflammatory depending on their states, i.e., while M1 macrophages are pro-inflammatory, M2 cells are anti-inflammatory. Specifically, M1 macrophages are activated during inflammatory response to eliminate environmental offenses by releasing ROS and activating pro-inflammatory T cells. Net mitochondrial free radicals can stimulate the production of pro-inflammatory cytokines such as IL1β by activating NFқB, a redox-dependent TF, or through the production and stabilization of GSH; and cleaves IL1β into its active form by releasing TRX1 towards the activation of leucine-rich repeat pyrin domain containing 3. These cytokines, once being released to the extracellular milieu, condition the activity of other bystander cells and cause inflammation that, in turn, activate M1 macrophages towards more ROS production 25 (**Figure 3**). In addition, M1 macrophages suppress the anti-inflammatory response under low oxygen conditions by producing nitric oxide (NO) that down-regulates the expression of forkhead box P3 (FOXP3), a TF capable of promoting the expression of anti-inflammatory regulatory T cells (Tregs); and M2 macrophages play an opposite role and exert an inhibitory activity on M1 cells.

Therefore, macrophages, being the dominating producers of RONS, are prone to oxidative modifications regarding their polarization and functionalities. As redox reactions represent a vital part of the physiological and pathological repertoire of macrophages, redox disequilibrium may easily reshape the constitution structure of macrophages toward sustained inflammation.

## Autoimmune disorder or cancer: consequences of pathological inflammation

The goal of inflammation, under the physiological state, is to replace injured tissues with healthy and regenerated tissues, intrinsically connecting it with cell proliferation. Yet, diseases may occur once cells proliferate in an uncontrollable manner and run into the pathological state.

Inflammation and proliferation are two critical and adjacent stages during wound healing (**Figure 1**). Deregulated transition from the inflammatory to the proliferative state is associated with the pathogenesis of many diseases.

As pro-inflammatory cytokines are excessively produced and macrophages intend to be attracted at the M1 state in response to redox disequilibrium, prolonged redox stress may sustain inflammation towards uncontrolled proliferation.

Like the inflow and outflow of water, cell proliferation and programmed cell death control the two ends of cells. While inflammation triggers uncontrolled cell proliferation, programmed cell death determines cell fate when cells are attracted into the inflammatory state. Specifically, cells are quickly updated, easily leading to autoimmune disorders that are typically featured with rapid cell growth and abnormal cell death, and become malignant when their decease paths are completely blocked, displaying another critical cancer hallmark, i.e., 'evading programmed cell death' (**Figure 3**).

Example autoimmune disorders include, e.g., rheumatoid arthritis, inflammatory bowel disease, type I diabetes mellitus, multiple sclerosis, Guillain-Barre syndrome, chronic inflammatory demyelinating polyneuropathy, myasthenia gravis, psoriasis, scleroderma, Graves' disease, Hashimoto's thyroiditis, vasculitis, and systemic lupus erythematosus, depending on the organ where the immune system attacks. Cancers can be classified by the loci where the initial malignancy occurs or grouped by the molecular patterns that capture their developmental paths. Focusing on skin disorders and using psoriasis and melanoma as the examples, we explain the role of cell death in stratifying diseases initiated from the same loci into autoimmune disorders and cancers, and how cytokines are solicited by excess net mitochondrial ROS and dictated by abnormal M1 macrophage polarization to promote cell growth as a result of redox disequilibrium.

Psoriasis is a skin autoimmune disorder caused by chronic inflammation and characterized by significantly reduced number of apoptotic cells than normal epidermis of healthy individuals 26. Multiple inflammatory cytokines including IL17, IL23, TNFα, IFNγ, IL1β, IL6, IL22, IL26, IL29 and IL36 have been reported to mediate the pathogenesis of psoriasis 27, with the IL23/IL17 axis and TNFα signaling playing the most essential roles 28. Specifically, keratinocytes release self-nucleotides and anti-microbial peptides such as LL37 to form self-nucleotides-LL37 complexes in response to external insults, which activates myeloid dendritic cells by acting on Toll-like receptors in plasmacytoid dendritic cells. Myeloid dendritic cells produce pro-inflammatory cytokines such as TNFα, IL12 and IL23 to stimulate the activities of Th1, Th17 and Th22 cells, and release additional cytokines such as IL1, IL17, IL22. IL17 stimulates the proliferation of keratinocytes and produces TNFα to recruit immunocytes that forms a positive feedback loop 29.

Therefore, attenuating inflammation has been proposed as an effective strategy for treating psoriasis and, accordingly, inhibitors of IL17, IL23 and TNFα such as secukinumab, ustekinumab and infliximab have been launched in clinics for psoriasis treatment 30, 31. Signalings known to drive the initiation and progression of psoriasis are canonical oncological pathways fostering uncontrolled cell proliferation such as mitogen-activated protein kinase (MAPK) or triggering cancer-associated inflammation such as NFκB and JAK/STAT (Janus kinase-signal transducers and activators of transcription). These pathways are redox sensitive 32. ROS can activate the MAPK cascades by activating receptors such as apoptosis signal-regulating kinase 1 (ASK1). While reduced thioredoxin binds to the N-terminal of ASK1 to inactivate it in the absence of oxidative stress, oxidized thioredoxin is dissociated from ASK1 that allows it to oligomerize, autophosphorylate, and become activated when exposed to the oxidative stress 33. ROS can activate or inhibit the NFκB pathway by facilitating or inhibiting the degradation of IkappaB (IκB) through modulating its upstream kinases such as IkappaB kinase (IKK), or by controlling the expression of NFκB-regulated genes through modulating the nuclear translocation of the NFκB heterodimers 34. ROS often activates NFκB signaling in the cytoplasm, but inhibits NFκB activity in the nucleus 35. ROS can trigger the formation and nucleus translation of STAT1-STAT3 dimer by activating JAK2 and tyrosine kinase 2 (TYK2), which leads to the expression of genes under JAK/STAT regulation 32.

Melanoma cells also express a variety of cytokines and chemokines that vary during disease progression. While the expression of IL6 and IL8 is associated with early melanoma, TGFβ, IL1 and GM-CSF are highly secreted by advanced melanoma cells, with TGFβ being diagnostic of patient metastasis 36. Keratinocytes release IL1, IL3, IL6 and IL8, and secrete TNFα and GM-CSF to adapt the tumor microenvironment to a condition favorable for melanocyte proliferation and tumorigenesis 37. In addition, cytokines and chemokines such as IL6 and IL8 and CXCL1 participate in the crosstalk between cancer-associated fibroblasts and melanoma cells through paracrine signaling 38.

The fact that psoriasis shares some signaling profiles with melanoma and, in particular, pathways driving uncontrolled cell proliferation and inflammation, enlightens us to propose the following hypothesis. Both psoriasis and melanoma are inflammation-driven disorders, and featured by fast cell growth and abnormal cell death. One giant discrepancy between both diseases lies in 'cell death'. While psoriasis has reduced cell death, programmed cell death in melanoma totally runs out of control (i.e., evading death signals). Thus, can we consider psoriasis a disease having acquired several hallmarks of cancers (i.e., uncontrolled cell proliferation, chronic inflammation), and become cancerous once the genome integrity is further interrogated? That is to say, psoriasis has already harbored some mutations on pivotal genes controlling cell growth, and may progress into skin cancer if additional oncogenes or tumor suppressors controlling cell death were mutated. If this hypothesis held true, can drugs for skin cancer treatment be applicable to psoriasis and vice versa? On the other hand, we may assume therapeutics targeting inflammation, alone or in combination with other agents, function well in treating both psoriasis and skin cancer.

## Cold atmospheric plasma: possible cure of autoimmune disease and cancer via attenuating inflammation

Redox modulation has emerged as a promising approach in treatments for various diseases. This strategy hinges on manipulating the redox states within cells, which are crucial for numerous cellular processes and can influence disease progression. There are some approaches to redox modulation that can be employed in the treatment of these diseases, such as CAP, Photodynamic Therapy (PDT) and nanotechnology-based therapies 39-44. In this section, we will use CAP as an example to illustrate its potential as a cure for autoimmune disorders and cancers through the attenuation of inflammation.

### Cold atmospheric plasma conveys medical use by attenuating inflammation

CAP, being the fourth state of matter, is partially ionized plasma composed of various RONS including short-lived species such as ‧OH, ‧O_2_^-^, ‧NO, and long-lived species such as ozone (O_3_) and hydrogen peroxide (H_2_O_2_) 45. It has been widely used in many areas of the medical sector, e.g., hemostasis 46, sterilization 47, disinfection 48 and, importantly, anti-inflammation 49. CAP has also been found to selectively kill cancer cells without harming their healthy peers, namely 'selectivity against cancers' 50. Though different CAP ejection sources and parameter configurations were used, consecutive documents have been reported on the success of using CAP in treating various types of cancers such as triple negative breast cancer 51, prostate cancer 52, bladder cancer 53, pancreatic cancer 54, liver cancer 55, and melanoma 56. In clinics, CAP has been succeeded in securing the life of a 75-year late-stage pancreatic cancer patient in 2016, and curing a 33-year old rare relapsed incurable peritoneal sarcoma patient in 2019 57. The first clinical trial approved by FDA (NCT4267575) employing CAP as an onco-therapeutic approach saved the lives of 17 out of 20 late-stage solid cancer patients in 2021 58. However, CAP has not been largely launched to the bedside due to the lack of guidelines on its dose determination and 45, importantly, consensus on the molecular mechanisms driving its anti-cancer efficacy of CAP.

To accelerate the rate of translating CAP into clinics as an oncotherapy, a plethora of working mechanisms have been proposed including, but are not limited to, inducing apoptosis/ferroptosis 59, 60, arresting migration/invasion 60, causing metabolic reprogramming 61, triggering anti-tumour immune response 62. The most plausible theory, so far, states that CAP functions by imposing cells with redox stress that exceeds the death threshold of cancer cells whereas that of normal cells can easily adapt to the normal state given their intact antioxidant system 63. Though this theory can well explain the selectivity of CAP against the bulk tumor cells as cancer cells typically have a higher basal redox level than normal cells 64, it becomes helpless when CAP was demonstrated to selectively kill cancer stem cells that have more robust antioxidant network than normal cells.

Given the intrinsic connections between redox homeostasis and inflammation, and the redox nature of CAP, we believe that it is the anti-inflammation nature of CAP that enables its multifaceted medical utilities. Therefore, we foresee an extension of miracles CAP so far have demonstrated in the medical sector to all pathological manifestations driven by prolonged inflammation. Below, we summarize the two extreme conditions caused by chronic inflammation and subjected to redox regulation, and use psoriasis and melanoma as the example pathological conditions to support our hypothesis with scientific evidence.

### Cold atmospheric plasma plays bidirectional roles in maintaining redox homeostasis

Redox disequilibrium can be caused by both oxidative and reductive stresses. As the counterpart of oxidative stress, reductive stress can occur in response to conditions that shift the balance of the redox couples such as GSH/GSSG and Cys/Cyst to a more reducing state. Over-expression of components in the antioxidant enzymatic system or excess production of reducing equivalents may induce alterations in the formation of disulfide bonds among proteins that lead to modified signaling pathways and transcriptome towards reduced mitochondrial function and decreased cellular metabolism. Chronic reductive stress may, in turn, induce oxidative stress that, again, stimulates reductive stress, forming a feedback loop. This eventually contributes to the development of many inflammation-driven diseases such as rheumatoid arthritis and cancer through, e.g., abnormal NFқB signaling 65.

**Figure 4:**
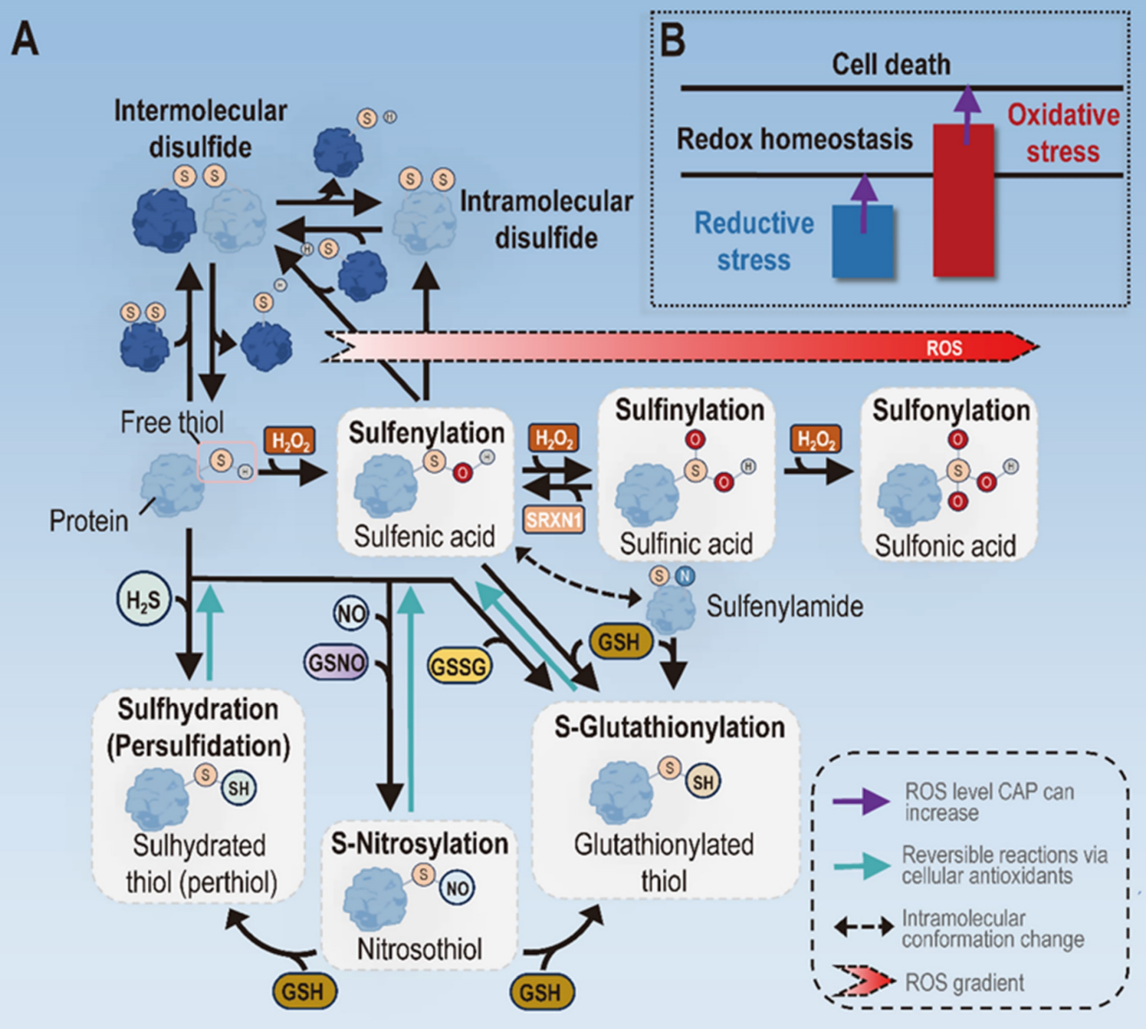
** Types of mechanisms driving the role of CAP in attenuating inflammation. (A)** Molecular bases on mechanisms CAP may adopt to attenuate inflammation. Different types of oxPTMs may occur to the thiol group of the cysteine amino acids in proteins that lead to altered protein structures and functionalities. One important thiol oxPTM is the formation of disulfide bonds, both intermolecularly and intramolecularly, which is known to activate or inhibit the function of target proteins. Another series of thiol oxPTMs include sulfenylation, sulfinylation and sulfonylation that occur with increased levels of H_2_O_2_. Under low H_2_O_2_ presence, free thiols undergo sulfenylation to form sulfenic acids that mediate redox signaling. With increased oxidative stress, sulfenic acids undergo sulfinylation to form sulfinic acids, the process of which can be reversed in the presence of SRXN1. When the oxidative level goes even higher, sulfinic acids undergo further reactions via sulfonylation to form sulfonic acids, the final irreversible form of thiols. Other modifications of free thiols include the sulfhydration by H_2_S, S-nitrosylation induced by reaction nitrogen species, and S-glutathionylation in the presence of GSH. **(B)** Mechanisms CAP may adopt to attenuate inflammation. CAP may attenuate inflammation by removing the reductive stress under low doses or triggering cell death under high doses. Panel A is redrawn and adapted under the terms and conditions of Creative Commons Attribution (CC-BY) license from 66.

CAP attenuates inflammation via maintaining redox homeostasis through two mechanisms, i.e., removing the reductive stress by directly elevating cellular oxidative level, or triggering the death of cells already under oxidative stress by imposing them with additional oxidative pressure. In addition, ROS, especially H_2_O_2_, can act as a second messenger to relay or augment signals via creating oxidative post-translational modifications (oxPTMs) on the thiol group of the amino acid cysteine of ROS-sensitive signaling molecules such as phosphatases (**Figure 4**).

The induction of oxPTMs is vital in redox signaling, given that cysteines constitute only 2% of the cellular proteome but participate in most PTMs 66. By forming disulfide bonds, oxPTMs can alter protein conformation and function 67. For instance, the disulfide bond bridged between the α- and γ-subunits of ATP synthase via Cys294 and Cys103 reduced ATP production 68. There are two forms of cysteine oxPTMs, i.e., disulfide bonds and sulfenylation (S-OH). Disulfide bonds can occur inter-molecularly and intra-molecularly between thiyl radicals (RS•) of two adjacent free thiols 66. S-OH can react with free thiols of a target protein to form disulfide bonds, or be converted to RS• in the presence of reduced GSH to form disulfide bonds with other thiolates 69 (**Figure 4**). Another series of thiol oxPTMs include sulfenylation, sulfinylation and sulfonylation that occur with increased levels of cellular oxidative stress. Specifically, free thiols undergo sulfenylation to form sulfenic acids to mediate redox signaling under low ROS level, proceed further to form sulfinic acids via sulfinylation when the ROS level increases, and form the final irreversible form of thiols, i.e., sulfonic acids, via sulfonylation when the oxidative level goes even higher (**Figure 4**). As a reversible process of oxPTM, the existence of S-glutathionylation allows cells with the plasticity of coping with redox stress and signaling 69. It is worth noting that oxPTM-mediated signaling occurs under low cellular ROS level, and excessive redox pressure may lead to deleterious consequences by forming irreversible sulfonic acids and, ultimately, loss of function of important proteins in the cell 70. This not only highlights the detrimental effects of increasing oxidative stress in cells, but also explains the molecular basis of cell death triggered by redox stress.

### Cold atmospheric plasma for treating autoimmune disorders: psoriasis as an example

The pathogenesis of autoimmune diseases is associated with a consequence of intense episodes of inflammation 71. Incremental evidence has demonstrated the efficacy of CAP in treating autoimmune disorders such as psoriasis, vitiligo, and rheumatoid arthritis 44, 72-81. Ding C *et al.* demonstrated that *in vivo* treatment with CAP could reduce inflammation and proliferation of synovium in adjuvant-induced arthritis rats, inhibit blood flow, and improve oxidative stress indicators. In addition, *ex vivo* CAP treatment reduced cell viability, invasion, and migration of rheumatoid arthritis fibroblast-like synoviocytes (RA-FLS) 73. These findings suggest that CAP could have a positive effect on rheumatoid arthritis by affecting cell survival and apoptotic pathways​​. Zhai S *et al.* explored the effectiveness of CAP-activated hydrogel in treating vitiligo 77. Below we take psoriasis as an example to discuss the possible medical use of CAP in treating autoimmune disorders.

Psoriasis is a typical pathological state of the skin as a result of chronic oxidative stress. Excessive ROS production may damage DNA, proteins and lipids, leading to the secretion of pro-inflammatory cytokines, over-activated NFκB and MAPK signalings, hyper-stimulated Th1/Th17 cells 82 and, importantly, reduced expression of programmed cell death ligand 1 (PD-L1) 83. It is worth noting that administrating mice carrying psoriasiform dermatitis with the recombinant PD-L1 effectively removed psoriatic skin symptoms 84.

It was reported that macrophages were primed to favor M1 to M2 among psoriasis carriers 85, and treating the patients with TNF-α inhibitors could reduce the disease activity by attenuating the M1 phenotype 86. *In vivo* evidence from murine models suggested that treating psoriatic lesions with a TLR7 agonist could shift macrophages to a higher M1/M2 ratio, where excessive stimulation of TLR7 was associated with psoriasis pathogenesis 87. In an *in vitro* study, the transcript level of the gene encoding RGC-32, a protein actively participating in M2 macrophage polarization, was reduced in psoriatic lesions, leading to skewed macrophage polarization toward the M1 state 88. Thus strategies capable of restoring the abnormal M1/M2 ratio have been established and believed to be promising. For instance, IL35, a known anti-inflammatory cytokine 89, 90, has been shown effective in treating psoriasis by tilting the M1/M2 ratio to favor the M2 phase both *in vitro* and *in vivo* 91.

Presently, CAP has been applied to treat two refractory psoriasis subtypes, i.e., palmoplantar psoriasis and inverse psoriasis, with great success, extending the possible medical application scenarios of CAP to autoimmune disease treatment 75, 78. By stimulating the local inflammatory micro-environment of psoriasis in HaCaT cells using LPS/TNFα and generating mice carrying psoriasiform dermatitis using imiquimod, Gan *et al.* found that plasma-activated medium (PAM) selectively inhibited hyper-proliferative keratinocytes by reducing cytokines TNFα, IL17, IL22, suggestive of the anti-inflammatory role of CAP in treating psoriasis and enhanced sensitivity of inflammatory cells to CAP treatment 74. Consistent with this finding, Lee *et al.* showed the inhibitory role of CAP on the expression of psoriasis-related cytokines and chemokines IL6, IL17, IL22, CCL20, CXCL1, and suppression on Th17 cell differentiation using a psoriasis mice model; also in this study, CAP was found capable of enhancing PD-L1 expression in HaCaT cells, suggestive of the role of CAP in counteracting T cell over-activation 76. To enable sufficient and prolonged CAP supply to the lesion area, Kim *et al.* fabricated a CAP patch to alleviate the psoriatic symptoms by restoring the normal differentiation of Keratin 1 without specific toxicity, and reducing the secretion of inflammatory cytokines and chemokines IL1β, IL6, IL8, CCL17 and CCL22 80. Controversially, Zhong *et al.* reported the role of CAP in inducing the expression of pro-inflammatory cytokines and chemokines IL6, IL8, TNFα, IL1β, IL10, IFNγ as well as reducing the expression of anti-inflammatory IL12 in human keratinocyte HaCaT cells that, ultimately, leads to reduced keratinocyte proliferation as a result of mitochondrial dysfunction and lysosome leakage caused by excessive redox stress 79.

### Cold atmospheric plasma for treating cancers: melanoma as an example

Tumor-associated inflammation is one hallmark of cancer 1. Being the most life-threatening and frequently reported type of skin cancers sensitive to CAP treatment, melanoma is originated from uncontrolled proliferation of melanocytes where multiple signaling cascades controlling cell growth and inflammation such as MAPK, phosphatidylinositol-3-kinase (PI3K)/AKT, NFκB and STAT pathways are activated 92, 93.

Mechanisms so far known to explain the efficacy of CAP in arresting the aggressiveness of cancers include the trigger of programmed cell death and attenuation of cancer-associated inflammation 94, 95. CAP can induce programmed cell death through inducing ROS and causing mitochondrial dysfunction in cancer cells 95. The anti-oxidant system of tumor cells is more fragile and susceptible to the influence of ROS than their healthy peers, inhibition of which leads to decreased GSH/GSSG ratio and perturbed mitochondrial redox homeostasis that, ultimately, trigger the death of malignant cells 64. CAP was demonstrated to selectively induce apoptosis via ASK1- and/or unfolded protein response (UPR)- mediated pathways in melanoma cells without harming that of normal melanocytes by imposing cells with additional ROS 96, 97. Mina *et al.* reported that CAP caused melanoma cell autophagy by increasing the mRNA expression of LC3 and ATG5 using B16 cells as the tumor model 98. In addition, CAP was shown capable of inducing immunogenic cell death (ICD) of melanoma cells and releasing tumor-associated antigens *in situ* towards stimulated anti-tumour immunity and, accordingly, a portable CAP device was established to prolong the survival of tumor-bearing mice by activating T cell-mediated anti-tumour immune response* in vivo* 99. Also, CAP suppressed cancer-associated inflammation by re-polarizing macrophages towards the M2 state or guiding M0 macrophage differentiation towards the M2 state to foster an anti-inflammatory environment, provided that M2 macrophages are more robust than M1 cells in tolerating mitochondrial stress 100.

## Conclusion

Through delineating the relationship between inflammation, redox equilibrium, macrophage polarization and the biochemical features of CAP, and taking two primary inflammation-driven diseases, i.e., autoimmune disorders and cancers, as the examples, we propose prolonged inflammation being the cause of many diseases including those exemplified. This is consistent with the fact that chronic inflammation is the sign of many autoimmune diseases and the cause of many precancerous lesions. Being a critical step in physiological inflammation, inappropriate inflammation-proliferation transition may position cells into a hyper-growth condition that, easily, leads to autoimmune disorders or cancers, the pathogenesis of which highly depends on the controllability of their death programs. Importantly, we propose the paramount role of mitochondrial redox equilibrium in preventing pathological inflammation and, consequently uncontrolled cell growth, which may be regulated by M0 differentiation, among other possible mechanisms of action. That is, cells are easily conditioned under a pro-inflammatory environment if M0 favored the M1 state, and under an anti-inflammatory condition if M2 macrophages dominated. As a redox modulatory tool, the molecular mechanism of CAP in selectively killing cancer cells has been positioned to its intrinsic efficacy in attenuating pathological inflammation. This has substantially extended the medical scenarios feasible for CAP application to encompass all inflammation-driven disorders including autoimmune diseases and possibly other pathological syndromes driven by chronic inflammation such as atopic dermatitis that has already been witnessed with evidence 101.

**Figure 5:**
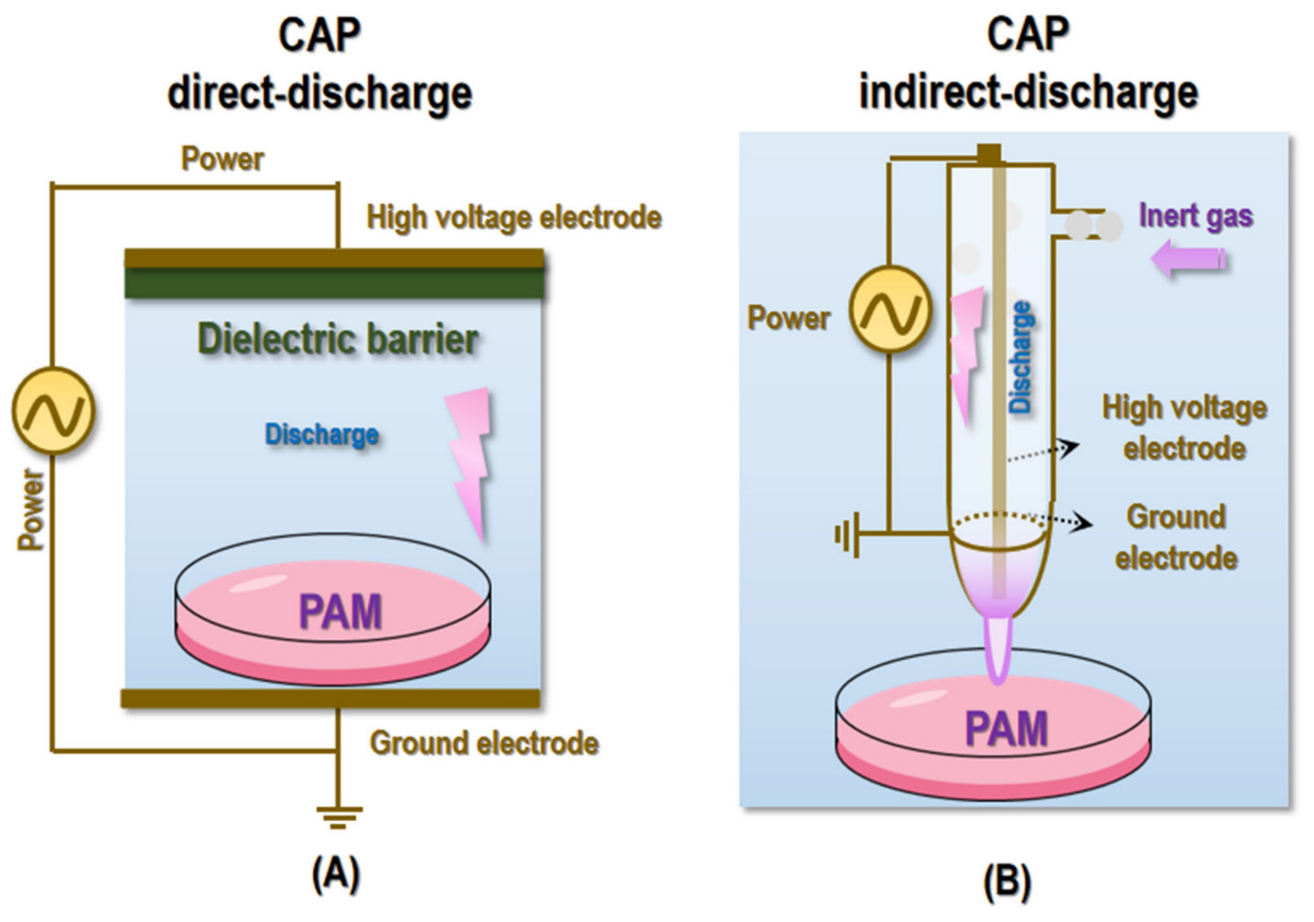
** Illustrative diagrams showing typical devices for CAP generation and PAM preparation. (A)** CAP direct-discharge and PAM preparation. **(B)** CAP indirect-discharge and PAM preparation.

It is worthwhile to mention that with CAP direct or indirect charge (**Figure 5**), it may be difficult to reach deep lesions regarding its clinical launch. Yet, CAP can be prepared in the form of other states such as liquid and semi-liquid to substantially expand the scenarios feasible for its medical use (**Figure 5**). Depending on the materials to be activated, CAP can be given different names such as PAM, plasma-activated water (PAW), plasma-activated Ringer's lactate solution (PAL) 102, and plasma-activated gel (PAG). Take PAM as the example, it has been widely used for cell culturing and *in vivo* animal model establishment in plasma associated investigations 103. One unique superiority of PAM or similar as compared with CAP discharge is that reactive species stored in it can be preserved for a relatively long time and be easily delivered to internal structures (through, e.g., injection, perfusion or rinse) for treating lesions that otherwise is difficult to reach. Advanced techniques also emerge that currently can store the active components of CAP in hydrogels for sustained release and reach deep tumors via localized injections 62. In addition, CAP was reported capable of inducing ICD and priming the immune response, rendering it possible to kill distantly located transformed cells or recurrent tumors. As one example, a portable air-fed CAP device (namely aCAP) was fabricated to manage post-surgical cancers with demonstrated efficacy in evoking a strong T cell-mediated immune response 104. Therefore, it is believed that with our incremental knowledge on the fundamental cause driving various pathogenesis and the mechanisms-of-action potentiating the biomedical utility of CAP, more advanced forms of CAP will become available, alone or in combination with other therapeutic modalities or techniques, to truly benefits people with the fourth state of matter. Last but not least is the dose-dependent nature of redox regulatory tools including CAP in human health that may play an opposite role if applied, unintentionally, with over-dosage 105, where radical scavengers such as ascorbate, carotenoids, spermidine and uric acid can be paired with CAP to achieve highly flexible regulations on cellular redox homeostasis.

By exemplifying psoriasis and melanoma, the two pathological conditions carefully selected to reflect the two extreme conditions caused by disrupted redox homeostasis, we uniquely identified 'chronic inflammation' as the fundamental cause urging the onset of various clinical syndromes and the shared role of disrupted 'redox homeostasis' in driving these diseases. In addition, insights provided here may revolutionize our concept in what CAP can do and also explain why CAP can demonstrate its various medical miracles (such as successes in treating psoriasis and melanoma) towards a unified consensus on its molecular mechanism. Through doing this, we believe that this paper can truly advance the biomedical field by marching towards systematic precision medicine.

Despite the great promise that redox regulatory tools such as CAP can make in human health, most supportive evidence are on the side of cancers. Given the diversified etiological causes and distinct pathological manifestations of autoimmune disorders, intensive effort is needed to explore the utility, molecular mechanism and application protocol of CAP in treating different kinds of autoimmune diseases.

## Abbreviations

ROS: reactive oxygen species
redox: reduction-oxidation
CAP: cold atmospheric plasma
TRX1: thioredoxin 1
Cys: cysteine
PTMs: post-translational modifications
NRF2: nuclear factor erythroid 2-related factor 2
TF: transcription factor
GPX4: glutathione peroxidase 4
ARE: anti-oxidant responsive element
CRE: cyclic AMP responsive element
SP-1: specificity protein-1
NFκB: nuclear factor kappa B
GSH: glutathione
GSSG: disulfide-bonded dimer
Cys/Cyst: cysteine/cystine
O_2_^-^: superoxide anion
RONS: reactive oxygen and nitrogen species
ACO: aconitase
KGDH: α-ketoglutarate dehydrogenase
TCA: tricarboxylic acid
ETC: electron-transport chain
PDH: pyruvate dehydrogenase
GPDH: glycerol-3-phosphate dehydrogenase
DHOH: dihydroorotate dehydrogenase
MAOA/B: monoamine oxidases A and B
B5R: cytochrome *b*_5_ reductase
aTCP: α-tocopherol
MnSOD: manganese superoxide dismutase
Cat: catalase
PGPX: phospholipid hydroperoxide glutathione peroxidase
GR: glutathione reductase
PRX3/5: peroxiredoxins 3/5
GRX2: glutaredoxin 2
TRXR2: thioredoxin reductase 2
‧OH: hydroxyl radical
^¹^O_2_: singlet oxygen
ONOO^-^: peroxynitrite
CXCL8: chemokine (C-X-C motif) ligand 8
IL1β: interleukin 1β
TNFα: tumor necrosis factor α
IFNγ: interferon γ
GM-CSF: granulocyte-macrophage colony stimulating factor
Th1: T helper 1
NLRP3: Nod-like receptor family pyrin domain containing 3
NO: nitric oxide
FOXP3: forkhead box P3
Tregs: regulatory T cells
MAPK: mitogen-activated protein kinase
JAK/STAT: Janus kinase-signal transducers and activators of transcription
ASK1: signal regulating kinase 1
IκB: IkappaB
IKK: IkappaB kinase
TYK2: tyrosine kinase 2
PDT: Photodynamic Therapy
O_3_: ozone
H_2_O_2_: hydrogen peroxide
oxPTMs: oxidative post-translational modifications
S-OH: sulfenylation
RS•: thiyl radicals
RA-FLS: rheumatoid arthritis fibroblast-like synoviocytes
PD-L1: programmed cell death ligand 1
PAM: plasma-activated medium
PI3K: phosphatidylinositol-3-kinase
UPR: unfolded protein response
ICD: immunogenic cell death
PAW: plasma-activated water
PAL: plasma-activated Ringer's lactate solution
PAG: plasma-activated gel
